# Multi-omics profiling reveals microbiota–gut–brain axis dysregulation in an LPS-induced model of sepsis-associated encephalopathy

**DOI:** 10.3389/fcimb.2026.1849929

**Published:** 2026-09-07

**Authors:** Qi Shan, Ying Yu, Yongjie Zhang, Yiming Li, Jianqiang Zhang, Li Zhang, Qiang Liu, Hong Fu, Zhifeng Qu, Jiajia Duan, Qizhi Fu

**Affiliations:** 1 Department of Intensive Care Unit (Internal Medicine), The First Affiliated Hospital, and College of Clinical Medicine of Henan University of Science and Technology, Luoyang, China; 2 Department of Laboratory Medicine, First Affiliated Hospital of Chongqing Medical University, Chongqing, China; 3 Department of Anesthesiology, The First Affiliated Hospital, and College of Clinical Medicine of Henan University of Science and Technology, Luoyang, China; 4 Department of Clinical Laboratory, The First Affiliated Hospital, College of Clinical Medicine of Henan University of Science and Technology, Luoyang, China; 5 Department of Rehabilitation, The Third People’s Provincial Hospital of Henan Province, Zhengzhou, China; 6 Department of Medical Equipment, The First Affiliated Hospital, and College of Clinical Medicine of Henan University of Science and Technology, Luoyang, China; 7 Luoyang Key Laboratory of Zebrafish Toxicology Research, Luoyang Key Laboratory of Transplantation and Immunological Studies for Haematological Diseases, and College of Clinical Medicine of Henan University of Science and Technology, Luoyang, China

**Keywords:** gut microbiota, metabolomics, microbiota-gut-brain axis, sepsis-associated encephalopathy, transcriptomics, zebrafish

## Abstract

**Background:**

Sepsis-associated encephalopathy (SAE) is a common complication of sepsis. Its underlying mechanisms remain incompletely understood, and effective treatments are still lacking. Recent studies have shown that the microbiota-gut-brain axis may play a key role in the pathogenesis of SAE, but its potential mechanisms have not yet been clarified.

**Methods:**

In this study, we used a lipopolysaccharide (LPS)-induced zebrafish endotoxemia model and systematically characterized microbiota–gut–brain axis-associated alterations using multi-omics profiling combined with histopathology, behavioral assessment, and blood–brain barrier integrity assays.

**Results:**

Our results showed that LPS exposure induced intestinal inflammation and barrier disruption, neurovascular dysfunction, anxiety-like behavior, and impaired cognitive function in zebrafish. Gut microbiota profiling revealed marked compositional alterations, accompanied by widespread metabolic disturbances in intestinal and brain tissues. Brain transcriptomic analysis showed that the differentially expressed genes were closely associated with the PI3K-Akt signaling pathway, cell adhesion, and autophagy-related pathways. Cross-omics association analyses suggested potential associations among gut microbial dysbiosis, metabolic disturbances, and brain molecular responses.

**Conclusion:**

These findings suggest that disruption of the microbiota–gut–brain axis may contribute to LPS-induced SAE-like neurobehavioral abnormalities and provide a basis for further mechanistic and therapeutic studies.

## 1 Introduction

Sepsis is defined as life-threatening organ dysfunction caused by a dysregulated host response to infection, remaining a leading cause of high morbidity and mortality in intensive care units worldwide (Evans et al., 2021; Singer et al., 2016). During the progression of sepsis, the brain not only participates in neural and immune regulation but is also one of the organs most vulnerable to injury (Sonneville et al., 2023). The incidence of sepsis-associated encephalopathy (SAE) in patients with sepsis is 50%–70%, and the associated mortality is high (Carter and Underwood, 2022). Even after recovery, these patients often experience serious sequelae, including cognitive impairment and affective disorders, which markedly compromise quality of life (Sekino et al., 2022). Because the pathogenesis of SAE has not been fully elucidated, and current clinical diagnosis still relies mainly on exclusion in the absence of specific biomarkers and targeted therapeutic options, the prevention and treatment of SAE remain major challenges in critical care medicine. Therefore, clarifying the mechanisms underlying SAE and developing effective preventive and therapeutic strategies are urgent priorities.

In recent years, the intestine has been regarded as the “engine” of sepsis and multiple organ dysfunction. The gut microbiota plays an important role in the pathophysiology of sepsis (Fay et al., 2017; Qian et al., 2025). Studies have shown that patients with sepsis exhibit gut microbial dysbiosis, marked alterations in microbial function, and metabolite profiles distinct from those of healthy individuals (Liu et al., 2019; Sun et al., 2023). These changes may reduce bacterial clearance capacity or aggravate dysregulation of the systemic inflammatory response in sepsis (Kim et al., 2020). Intensified systemic inflammation may further exacerbate neuroinflammation and thereby increase susceptibility to SAE (Fang et al., 2022). In addition, studies have suggested that the gut microbiota may influence delirium not only indirectly through inflammatory pathways but also through translocation of gut-derived bacteria to the brain, thereby affecting brain function (Garcez et al., 2023; Jiang and Tan, 2022; Singer et al., 2018). Moreover, animal studies have demonstrated that gut microbiota-derived short-chain fatty acids, such as acetate and butyrate, can markedly increase the levels of zonula occludens-1 (ZO-1) and occludin associated with BBB integrity and significantly suppress neuroinflammation by inhibiting the JNK and NF-κB signaling pathways (Singer et al., 2018; Wang et al., 2019). Therefore, exploring additional mechanisms by which the gut microbiota affects SAE will deepen our understanding of their relationship and provide a theoretical basis for the clinical diagnosis and treatment of SAE.

Zebrafish (*Danio rerio*) combine conserved vertebrate immunity, external fertilization, rapid development, advantages for live imaging, and abundant genetic tools, and have therefore been widely used in studies of inflammation, infection, and neurobehavior (Teame et al., 2019; Xie et al., 2020). In particular, transparent larvae and multiple fluorescent transgenic lines make it possible to track neutrophil recruitment, vascular leakage, and BBB function *in vivo* (Renshaw et al., 2006). These advantages make zebrafish a powerful model for investigating sepsis-associated gut-brain axis mechanisms and for high-throughput drug screening (de Abreu et al., 2019; Philip et al., 2017). On this basis, the present study established an SAE-like model by intraperitoneal injection of LPS in zebrafish, systematically evaluated intestinal inflammation/barrier injury, behavioral alterations, and neurovascular function, and integrated gut microbiome sequencing, multi-tissue metabolomics of the gut and brain, and brain transcriptomics for pathway enrichment and multi-omics analyses. Our aim was to depict the pathological phenotype of the LPS-induced gut-brain axis at a systems level and to provide clues for mechanistic and translational studies of SAE.

## 2 Methods

### 2.1 Zebrafish strains and husbandry

Adult wild-type (WT, AB) zebrafish were provided by the National Zebrafish Resource Center (Wuhan, China). Fish were maintained in a recirculating water system at 28 °C under a 14 h/10 h light/dark cycle and were fed three times daily. For embryo production, zebrafish were paired at a ratio of 1:1 overnight and separated with a divider. The divider was removed the next day, and spawning occurred within 1 h of the onset of the light cycle. Embryos were placed in 10-cm dishes containing 1× E3 solution supplemented with methylene blue (0.3 ppm) and maintained at 28.5 °C in an artificial climate incubator under a 14 h/10 h light/dark cycle until experimental treatment. The experimental animal use license number was SYXK (Zhe) 2022-0004. Animal housing and management complied with the requirements of AAALAC International accreditation (accreditation No. 001458), and the study was approved by the IACUC (approval No. IACUC-2023-7407-01).

### 2.2 Experimental design

Three independent experimental cohorts were established for different experimental purposes. Cohort 1 comprised four adult zebrafish groups: the control group (PBS), low-dose LPS group (1 μg/g), medium-dose LPS group (5 μg/g), and high-dose LPS group (10 μg/g). Three zebrafish from each group were used for intestinal inflammatory and mucosal barrier-related gene-expression analysis (n = 3 per group) to determine the appropriate LPS dose.

Cohort 2 consisted of 46 adult zebrafish divided into a control group and a high-dose LPS group, with 23 fish per group. Fish in each group were distributed among three tanks containing 10, 10, and 3 fish, respectively. This cohort was used to evaluate behavioral alterations, brain and intestinal histopathology, brain weight, intestinal length, transcriptomic profiles, metabolic alterations, and intestinal microbiota composition following treatment with the selected LPS dose of 10 μg/g.

Cohort 3 comprised two independent larval experiments using transgenic zebrafish lines. In each experiment, 60 larvae were divided into a control group and an LPS group, with 30 larvae per group and three replicate wells containing 10 larvae each. Tg(mpx) larvae were used to evaluate neutrophil recruitment, whereas Tg(fli1) larvae were used to assess vascular leakage after exposure to 100 μg/mL LPS. All zebrafish were randomly allocated to the indicated treatment groups.

### 2.3 LPS-induced zebrafish sepsis model

Adult zebrafish of the same age and with a similar growth status (>3 months old) were housed in tanks at three fish per tank. For injection, adult fish were anesthetized by immersion in tricaine (160 μg/mL). After anesthesia, different concentrations of LPS solution (1 μg/g, 5 μg/g, and 10 μg/g) were intraperitoneally injected using a microsyringe, whereas the control group received intraperitoneal injection of 10 μL PBS. All groups were injected once every 2 days until 7 days post-treatment (dpt).

### 2.4 Determination of inflammatory cytokines and cell junction genes

At 7 dpt, three zebrafish were selected from each of the four groups. The intestines were dissected and homogenized. RNA was extracted according to the kit instructions, reverse-transcribed to cDNA, and used for quantitative analysis of pro-inflammatory genes (*il-1β, il-6, il-12*, and *tnf-α*), anti-inflammatory genes (*il-10* and *tgf-β*), and intestinal mucosal barrier-related genes (*occludin, muc2.1, tjp2α*, and *zo-1*).

Total RNA was extracted using a universal RNA extraction kit (Wuxi Puhe, M004), and 1 μg of total RNA was used for cDNA synthesis. Reverse transcription was performed using a cDNA synthesis kit containing thermolabile double-stranded DNase (Biosharp, BL696A) to remove genomic DNA and synthesize cDNA. Primers used for RT-qPCR were designed and synthesized using Primer Premier 5.0 software, with *Gapdh* as the housekeeping gene (**Supplementary Table 1**). Reaction mixtures were prepared according to the RT-qPCR system. After amplification, results were automatically analyzed using a real-time quantitative PCR instrument, and relative gene expression differences between the control and experimental groups were calculated using the 2^-ΔΔCT method (Livak and Schmittgen, 2001).

### 2.5 Behavior test

#### 2.5.1 Open-field test

The open-field test was used to evaluate locomotor activity and anxiety-like behavior in adult zebrafish. Ten zebrafish were selected from each group for behavioral testing. The test was performed in an acrylic tank measuring 40 cm × 40 cm × 20 cm, which was divided into an inner zone (20 cm × 20 cm) and an outer zone. At 7 dpt, each zebrafish was individually placed in the center of the tank and allowed to acclimate freely for 5 min without external stimulation. Behavioral activity was subsequently recorded for 10 min using a behavioral tracking system, and the swimming time and swimming speed in the inner and outer zones were quantified and compared to assess the effects of sepsis on brain function in zebrafish.

#### 2.5.2 Novel tank test

The novel tank test was used to evaluate anxiety-like behavior in zebrafish. The tank was divided equally into lower, middle, and upper zones for independent analysis. At 7 dpt, one zebrafish was placed into the tank each time, and its behavior was immediately recorded for 10 min using a behavioral tracking system. The cumulative movement time in each zone and the latency to first enter the middle and upper zones (first ascent time) were analyzed.

#### 2.5.3 T-maze test

The T-maze test was used to assess spatial memory in zebrafish. T-maze memory training began at 4 dpt, with one fish trained at a time, three sessions per day, 5 min per session. In the T-maze, zone IV served as the reward arm and contained food, whereas zone III served as the punishment arm and was tapped when entered by the fish. During each training session, a zebrafish was first placed in zone I. After 1 min of free acclimation, the divider was removed to allow the fish to explore the maze freely for 5 min, and the fish was required to remain in zone IV for more than 30 s. If a fish failed to enter zone IV within 5 min, it was manually guided to that zone and kept there for more than 30 s. At 7 dpt, each fish was acclimated in zone I for 1 min, after which a 5-min video was recorded. The time taken for each fish to first reach zone IV and remain there for more than 30 s (or 300 s if not achieved within 5 min) was recorded.

### 2.6 Measurement of brain tissue weight and intestinal length

Ten zebrafish were dissected to obtain brain and intestinal tissues. Brain weights were recorded in milligrams using an electronic balance with a minimum readability of 1 mg, and intestinal length was measured with a ruler.

### 2.7 Hematoxylin and eosin staining

Five zebrafish were selected from each group. Brain and intestinal tissues were dissected and placed into 1.5-mL EP tubes. After being washed three times with enzyme-free PBS, the tissues were fixed in 4% PFA. After fixation, samples were dehydrated, embedded, sectioned, stained with hematoxylin and eosin (H&E), and scanned for analysis. Under light microscopy, neuronal arrangement, soma morphology, and inflammatory infiltration were evaluated in the brain gray matter, while villus height, luminal structure, and inflammatory cell infiltration were evaluated in the intestine.

### 2.8 Neutrophil counting

Tg(mpx:EGFP) zebrafish embryos at 6 h post-fertilization (hpf) were randomly selected and placed in culture dishes. At 72 hpf, the control group received no treatment, whereas the LPS group was exposed to 100 μg/mL LPS. After 6 h, ten zebrafish were randomly selected from each group, imaged under a fluorescence microscope, and the total number of green fluorescent neutrophils throughout the body was quantified using ImageJ (Schneider et al., 2012).

### 2.9 Vascular leakage assay

Tg(fli-1:EGFP) zebrafish embryos at 6 hpf were randomly selected and placed in culture dishes. At 72 hpf, the control group received no treatment, whereas the LPS group was exposed to 100 μg/mL LPS (Philip et al., 2017). After 6 h, ten zebrafish were randomly selected from each group for venous microinjection of 10 nL of 20 mg/mL rhodamine-conjugated dextran (Dextran-RB). After 30–60 min, fluorescence distribution inside and outside the cerebral vasculature was observed under a fluorescence microscope to assess vascular integrity.

### 2.10 Transcriptomic sequencing

Three adult zebrafish were collected from each group. Brain tissue was dissected in sterile dishes, immediately rinsed three times in ice-cold PBS, placed individually into sterile 1.5-mL EP tubes, and snap-frozen in liquid nitrogen. Samples were then subjected to RNA extraction, library construction, sequencing, and data analysis. First, total RNA was extracted from zebrafish tissues, and RNA integrity and concentration were assessed using a Qsep nucleic acid analyzer (BiOptic Inc.). After quality control, mRNA was enriched with Oligo(dT)-coated magnetic beads. Fragmentation buffer was then added to fragment the mRNA into short fragments. Using fragmented mRNA as the template, first-strand cDNA was synthesized with random hexamer primers, followed by second-strand cDNA synthesis using buffer, dNTPs, RNase H, and DNA Polymerase I. The cDNA was purified using a QIAQuick PCR kit and eluted with EB buffer. The purified double-stranded cDNA was then subjected to end repair, adenylation, and adapter ligation, followed by agarose gel electrophoresis to recover fragments of the desired size and PCR amplification to complete library preparation. After library quality control, sequencing was performed on the BGI T7 platform. Raw reads were processed by removing low-quality sequences and adapter contamination to obtain high-quality clean reads, and all downstream analyses were based on clean reads. Sequencing quality metrics, including read counts, clean bases, GC content, Q20/Q30 values, and mapping statistics for each sample, are summarized in **Supplementary Table 7**. Differential expression analysis was performed using DESeq2 (Love et al., 2014), and differentially expressed genes (DEGs) were defined by |log2FC| ≥ 1 and p < 0.05, followed by clustering analysis, Gene Ontology (GO) annotation, and KEGG pathway enrichment analysis.

### 2.11 Untargeted metabolomic analysis

Six adult zebrafish per group were randomly selected, and brain tissue, intestinal tissue, and intestinal contents were collected separately and snap-frozen in liquid nitrogen. Samples were homogenized in pre-cooled extraction solvent, ultrasonicated, centrifuged, vacuum-dried, and reconstituted in 50% acetonitrile containing 2-chloro-L-phenylalanine as an internal standard. LC-MS analysis was performed using a Thermo Vanquish UHPLC system equipped with an ACQUITY UPLC^®^ HSS T3 column coupled to a Thermo Orbitrap Exploris 120 mass spectrometer operating in positive and negative ESI modes. Raw data were converted to mzXML format using ProteoWizard (Kessner et al., 2008) and processed using XCMS for peak detection (Smith et al., 2006), retention-time correction, and alignment. Features with an RSD > 30% in QC samples were excluded (Want et al., 2013). PCA, PLS-DA, and OPLS-DA were performed using the R package ropls (Thévenot et al., 2015). PLS-DA model performance was evaluated by cross-validation using R²Y (cum) and Q² (cum). R²Y (cum) reflects the cumulative goodness-of-fit of the model, whereas Q² (cum) reflects its cross-validated predictive ability. Permutation testing was additionally performed to assess potential model overfitting. Differential metabolites were identified based on OPLS-DA-derived VIP values and univariate statistical testing, with VIP > 1 and a nominal p value < 0.05 as the predefined screening criteria. Differential metabolites were subsequently mapped to KEGG pathways for pathway enrichment analysis.

### 2.12 16S rRNA sequencing

Six zebrafish were randomly selected from each group for intestinal microbiota analysis. Under sterile conditions, the intestinal tissues were carefully dissected in sterile Petri dishes. The intestines were gently compressed, and the intestinal contents were collected separately into sterile 1.5-mL microcentrifuge tubes, with one sample placed in each tube. The samples were immediately snap-frozen in liquid nitrogen and subsequently stored at −80 °C until 16S rRNA gene sequencing was performed. Total microbial DNA was extracted from intestinal-content samples using the CretMagTM Power Soil DNA Kit. DNA quality and concentration were assessed by 1% agarose gel electrophoresis and NanoDrop2000. The V3–V4 region of bacterial 16S rRNA genes was amplified using the 341F/806R primer pair under the following PCR conditions: 94 °C for 2 min; 30 cycles of 94 °C for 30 s, 55 °C for 30 s, and 72 °C for 30 s; followed by 72 °C for 10 min. PCR products were recovered from 2% agarose gels, purified, and used for library construction with the VAHTS^®^ library preparation kit, followed by sequencing on the DNBSEQ-G99 PE300 platform. Raw data were quality controlled using fastp to remove low-quality reads, reads shorter than 50 bp, and reads containing N bases (Chen et al., 2018). Paired-end reads were merged using FLASH (minimum overlap 10 bp; mismatch rate ≤ 0.2) (Magoč and Salzberg, 2011), clustered into operational taxonomic units (OTUs) at 97% similarity using UPARSE with chimera removal, and taxonomically annotated against the Silva 138 database using the RDP classifier (threshold 70%) for subsequent bioinformatic analysis (Edgar, 2013; Wang et al., 20077). After quality filtering, all samples were rarefied to 40,550 sequences per sample for downstream analyses. Good’s coverage ranged from 99.978% to 100%, indicating sufficient sequencing depth (**Supplementary Table 8**). Alpha diversity indices (Chao1, ACE, Shannon, and Simpson) were first used to evaluate microbial diversity and richness, and intergroup comparisons were performed using the Wilcoxon rank-sum test. Beta diversity was evaluated based on the Euclidean distance matrix, and intergroup differences were assessed using the Wilcoxon rank-sum test. To further characterize microbial community composition, bar plots were used to display microbial distribution patterns in different groups, and linear discriminant analysis effect size (LEfSe) was applied to identify discriminatory dominant genera between the two groups (Segata et al., 2011).

### 2.13 Statistical analysis

Comparisons between two groups were performed using Student’s t test or the Mann–Whitney U test, whereas multiple-group comparisons were analyzed by one-way ANOVA. To minimize investigator bias, zebrafish were randomly allocated to the experimental groups or randomly selected for subsequent analyses, as appropriate. Data analysis was performed by investigators who were blinded to the group assignments. Data are presented as mean ± SD. Statistical analyses were conducted using GraphPad Prism 8.0.2, and p < 0.05 was considered statistically significant.

## 3 Results

### 3.1 LPS causes histopathological damage in zebrafish brain and intestinal tissues and promotes early neutrophil infiltration

**Figure 1:**
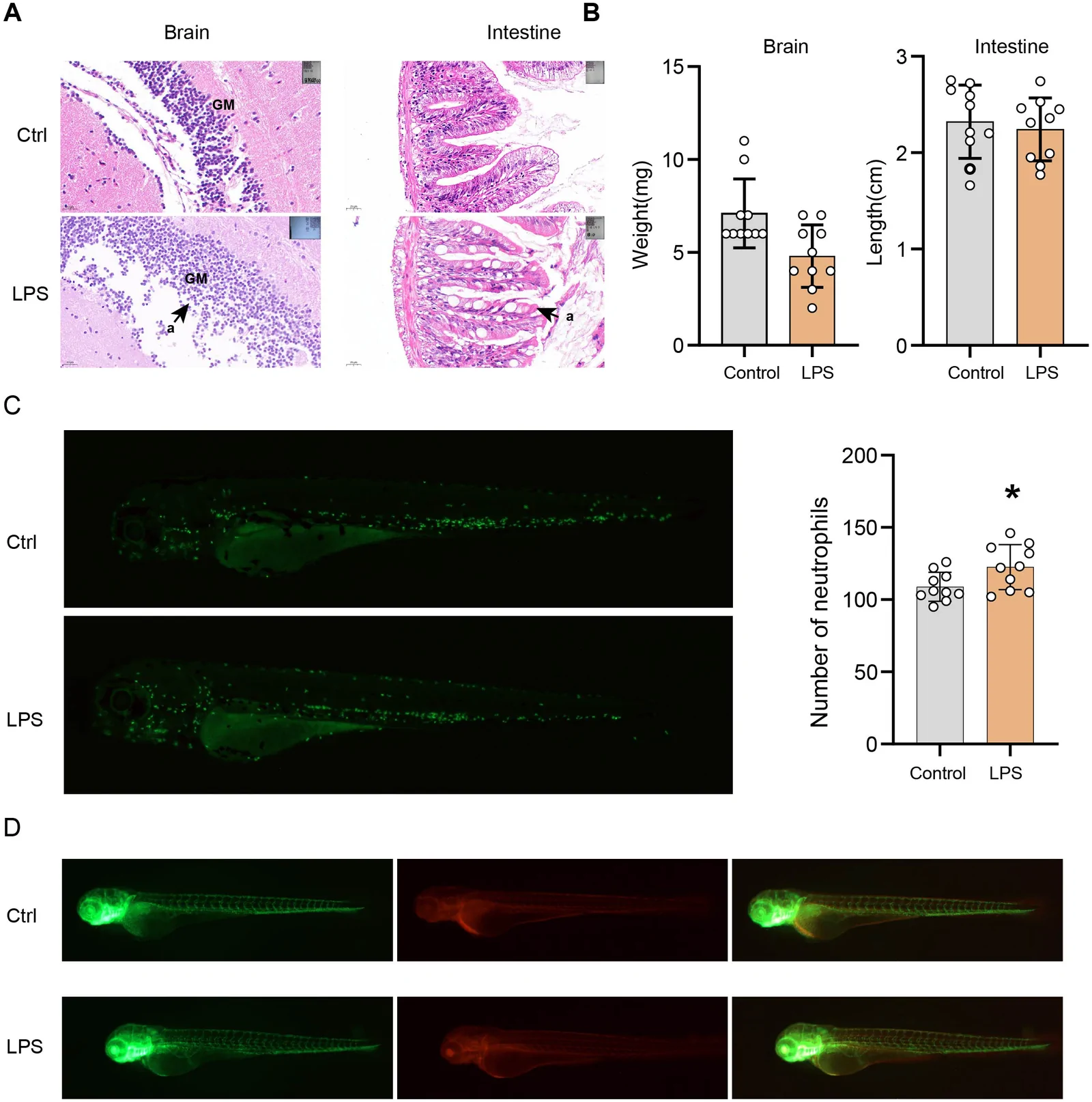
**(A)** H&E staining of brain and intestinal sections from adult zebrafish. Arrow “a” indicates widened intercellular spaces and loss of intestinal villi. GM, gray matter. **(B)** Effects of LPS-induced sepsis on brain tissue weight and intestinal length in zebrafish. **(C)** Fluorescence imaging and quantification of whole-body neutrophils in 72 hpf Tg(mpx:EGFP) zebrafish after 6 h of LPS exposure. **(D)** Fluorescence observation of vascular leakage in 72 hpf Tg(fli-1:EGFP) zebrafish after 6 h of LPS exposure. Statistical differences were analyzed by one-way ANOVA. *P < 0.05, **P < 0.01.

In this study, H&E staining of zebrafish brain and intestinal tissues was performed to evaluate the effects of LPS on these tissues. The results showed that LPS induced structural damage in both the brain and intestinal tissues of zebrafish. In brain tissue, neurons in the control group exhibited intact morphology, regular nuclei, and no inflammatory infiltration or edema. Compared with the control group, adult zebrafish in the LPS group showed reduced neuronal density in the gray matter and widened intercellular spaces (“a”) (**Figure 1A**). In intestinal tissue, the control group showed neatly arranged intestinal villi, clear crypt structures, and no obvious inflammatory infiltration. Compared with the control group, the LPS group exhibited loss of villi (“a” arrow) and massive neutrophil infiltration in the submucosa (**Figure 1A**). In addition, measurements of zebrafish brain weight and intestinal length showed that both brain weight and intestinal length were lower in the LPS group than in the control group, although the differences were not statistically significant (**Figure 1B**). In Tg(mpx:EGFP) larvae, 6 h of LPS exposure significantly increased the whole-body neutrophil count, especially with aggregation in the head and abdominal regions (p < 0.01) (**Figure 1C**). In Tg(fli-1:EGFP) larvae injected with Dextran-RB, fluorescence was mainly confined to the vascular lumen in the control group, whereas partial fluorescence leakage from cerebral vessels into surrounding tissues was observed in the LPS group (**Figure 1D**).

### 3.2 LPS induces intestinal inflammation and mucosal barrier injury in zebrafish

**Figure 2:**
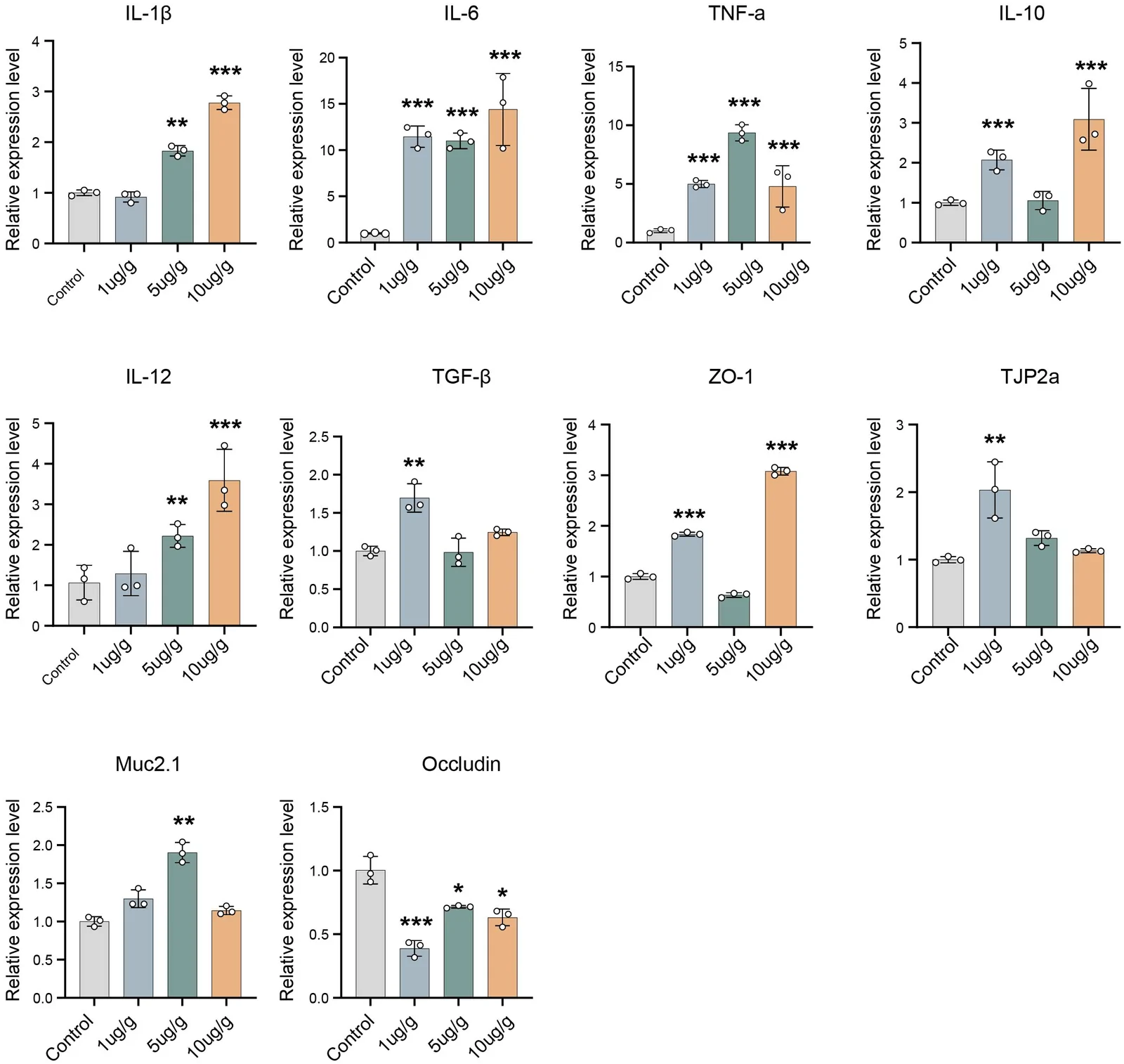
RT-qPCR analysis of the expression levels of pro-inflammatory genes (il-1β, il-6, il-12, and tnf-α), anti-inflammatory genes (il-10 and tgf-β), and intestinal mucosal barrier-related genes (occludin, muc2.1, tjp2α, and zo-1). *P < 0.05, **P < 0.01, ***P < 0.001.

We first used RT-qPCR to evaluate intestinal inflammation and barrier function in the control group and the low-dose LPS (1 μg/g), medium-dose LPS (5 μg/g), and high-dose LPS (10 μg/g) groups. As shown in **Figure 2**, compared with the control group, the low-, medium-, and high-dose LPS groups showed significantly increased expression of pro-inflammatory genes (*il-1β, il-6, il-12*, and *tnf-α*) and anti-inflammatory genes (*il-10* and *tgf-β*), indicating that LPS markedly induced an intestinal inflammatory transcriptional response. Meanwhile, expression of the tight junction protein occludin was significantly downregulated, whereas expression of the mucin gene *muc2.1* and the tight junction genes *tjp2α* and *zo-1* was upregulated, suggesting that LPS induced differential expression of intestinal barrier components. The high-dose LPS group exhibited the most pronounced changes in inflammatory and barrier-related genes and was therefore used for subsequent behavioral and omics analyses.

### 3.3 LPS induces anxiety-like behavior and cognitive impairment in zebrafish

**Figure 3:**
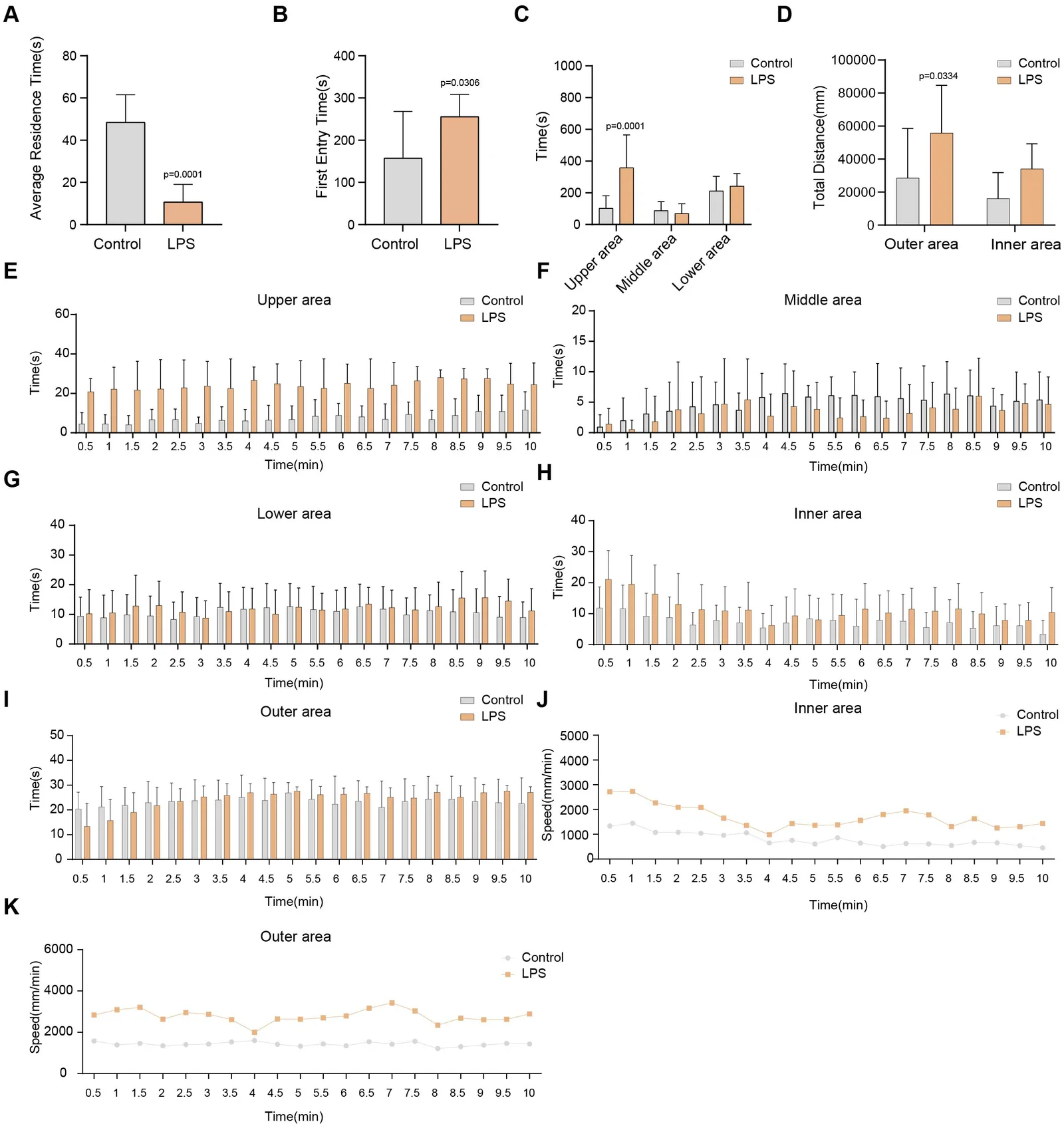
LPS induces anxiety-like behavior and impaired spatial learning and memory in adult zebrafish. **(A)** Mean residence time in the reward arm of the T-maze. **(B)** Latency to first enter the reward arm. **(C)** Cumulative residence time in the top, middle, and bottom zones in the novel tank test. **(D)** Total travel distance in the central and peripheral zones in the open-field test. **(E–G)** Segmented residence time in the top, middle, and bottom zones in the novel tank test. **(H, I)** Segmented residence time in the central and peripheral zones in the open-field test. **(J, K)** Segmented swimming speed in the central and peripheral zones in the open-field test.

Next, we assessed the effects of LPS on zebrafish neurobehavior through behavioral testing. T-maze results showed that, compared with the control group, the mean residence time in the reward arm was markedly reduced in the LPS group (48.50 vs. 10.71 s, p = 0.0001; Hedges’ g = −3.34), whereas the latency to first enter the reward arm was significantly prolonged (157.5 vs. 255.9 s, p = 0.0306; Hedges’ g = 1.10) (**Figures 3A, B**), indicating impaired spatial learning and memory following LPS exposure. In the novel tank test, compared with the control group, LPS-treated zebrafish spent significantly more time in the upper zone (358.7 vs. 102.5 s, p < 0.0001), whereas no significant between-group differences were observed in the middle or lower zones (p = 0.9636 and 0.8611, respectively) (**Figures 3C, E–G**). In the open-field test, compared with the control group, the LPS group traveled a significantly greater distance in the outer area (55,751 vs. 28,546 mm, p = 0.0334), whereas the difference in the inner area was not statistically significant (34,093 vs. 16,141 mm, p = 0.2014) (**Figure 3D**). The temporal profiles of residence time and swimming speed in the inner and outer areas are shown in **Figures 3H–K**. Taken together, these results indicate that LPS exposure was associated with impaired spatial learning and memory and alterations in exploratory and locomotor behavior in zebrafish.

### 3.4 Effects of LPS on gut microbial diversity in zebrafish

**Figure 4:**
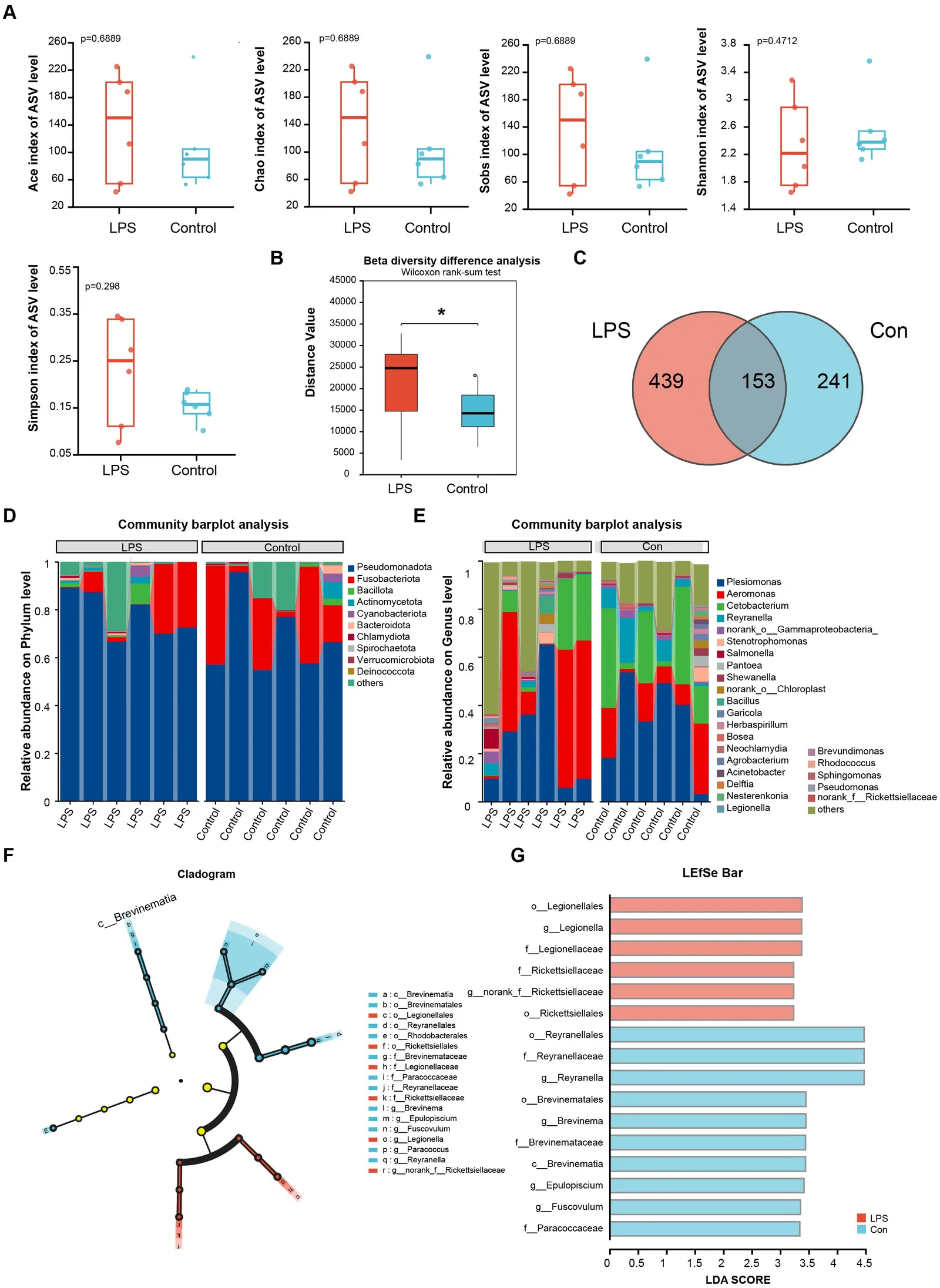
LPS treatment markedly altered the gut microbiota of zebrafish. **(A)** Alpha-diversity analysis of the gut microbiota in the LPS and control groups. **(B)** Beta-diversity difference analysis based on Euclidean distance. Statistical significance was assessed using the Wilcoxon rank-sum test. **(C)** Venn diagram of ASV distribution between the LPS and control groups. **(D)** Relative abundance of bacterial phyla in the LPS and control groups. **(E)** Relative abundance of bacterial genera in the LPS and control groups. **(F)** Multilevel taxonomic cladogram of the zebrafish gut microbiota in the LPS and control groups (from phylum to genus). **(G)** LDA histogram of the gut microbiota in zebrafish from the LPS and control groups; higher LDA scores indicate a greater contribution of a taxon to the differential effect, LEfSe analysis showing discriminative taxa across multiple taxonomic levels. The prefixes o: f: and g: indicate order, family, and genus, respectively. *P < 0.05.

To investigate whether the potential effects of LPS in zebrafish are regulated through the microbiota-gut-brain axis, gut content samples were collected from both groups, and changes in gut microbial structure were analyzed by high-throughput 16S rRNA gene sequencing. Analysis of 16S rRNA in intestinal contents showed no significant changes in alpha diversity indices, indicating that the richness and diversity of the microbial community were not substantially altered (**Figure 4A**). Beta-diversity analysis based on Euclidean distance showed a significant difference in community dissimilarity between the LPS and control groups (**Figure 4B**). The Venn diagram showed that 153 amplicon sequence variants (ASVs) were shared between the LPS and control groups, whereas 439 and 241 ASVs were unique to the LPS and control groups, respectively (**Figure 4C**). To determine LPS-induced changes in specific bacterial taxa, the gut microbiota composition of the two groups was first analyzed at the phylum and genus levels. At the phylum level, compared with the control group, the relative abundances of *Fusobacteriota* and *Actinomycetota* were decreased in the LPS group, whereas those of *Pseudomonadota* and *Bacillota* were increased (**Figure 4D**). At the genus level, compared with the control group, the relative abundances of *Plesiomonas, Aeromonas, Cetobacterium, unclassified_k:norank_d:Bacteria, unclassified_f:Rhizobiaceae, unclassified_f:Enterobacteriaceae*, and *Reyranella* were markedly altered in the LPS group (**Figure 4E**). Next, LEfSe analysis was performed to compare differential genera between the control and LPS groups (LDA > 2, p < 0.05) (**Figure 4F**). Differential taxa were identified across multiple taxonomic levels, including order, family, and genus. A total of 19 discriminative taxa were identified across different taxonomic levels, of which 13 were enriched in the control group, including the genera *Fuscovulum, Reyranella*, and *Paracoccus*, and 6 were enriched in the LPS group, including the family *Rickettsiellaceae* and *the genus Legionella* (**Figure 4G**; **Supplementary Table 2**).

### 3.5 LPS exposure induces metabolic pathway alterations in zebrafish brain tissue, intestinal tissue, and intestinal contents

**Figure 5:**
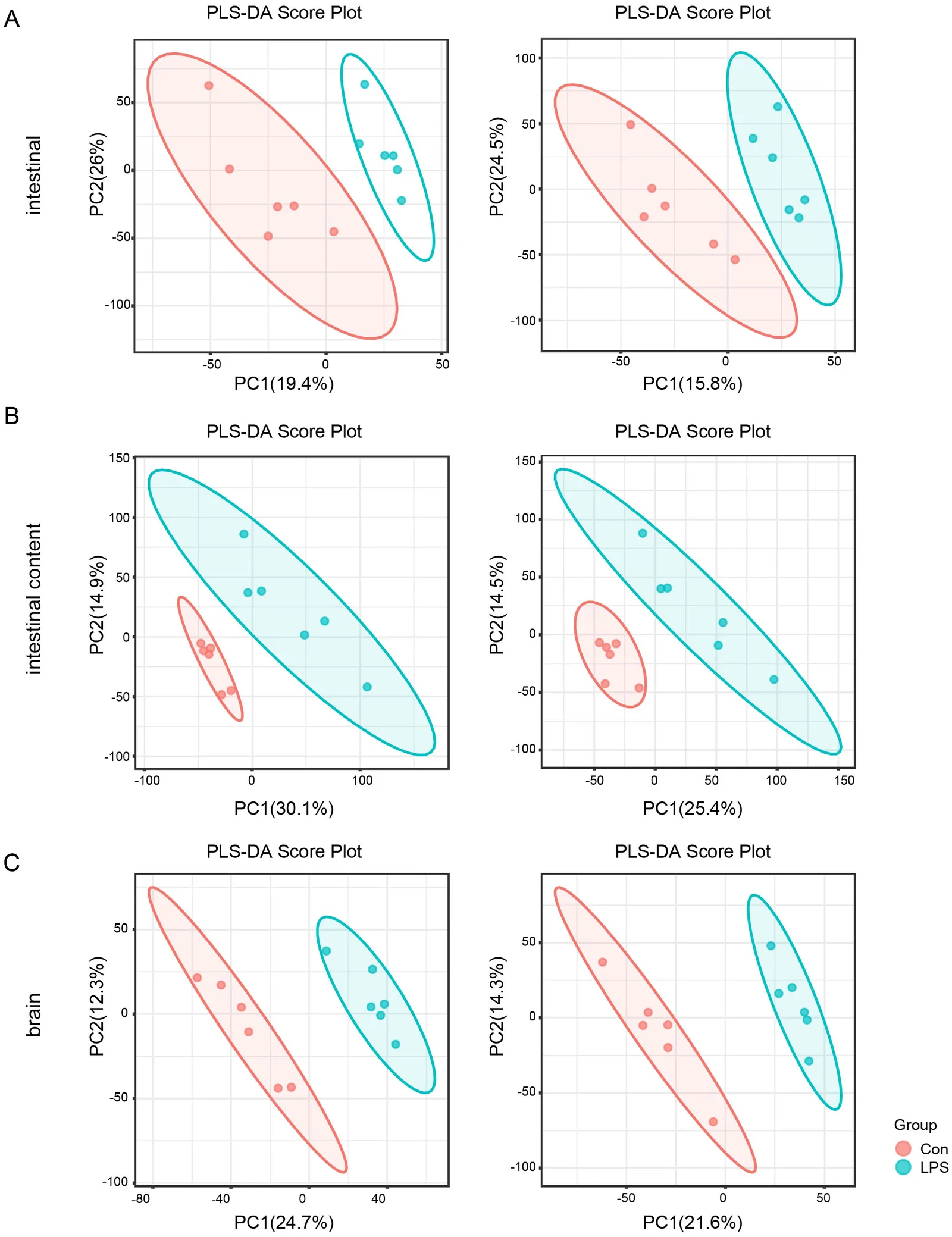
PLS-DA score plots of metabolomic profiles in intestinal tissue, intestinal content, and brain tissue from the control and LPS groups under positive and negative ion modes. **(A)** Intestinal tissue. **(B)** Intestinal content. **(C)** Brain tissue.

We systematically investigated the metabolic changes induced by LPS in zebrafish brain tissue, intestinal tissue, and intestinal contents. PLS-DA score plots showed clear separation between the LPS and control groups in intestinal tissue, intestinal contents, and brain tissue (**Figure 5**). The model-validation results supported the observed group separation without evidence of substantial overfitting. Differential metabolites were identified using the thresholds VIP > 1 and p < 0.05.

**Figure 6:**
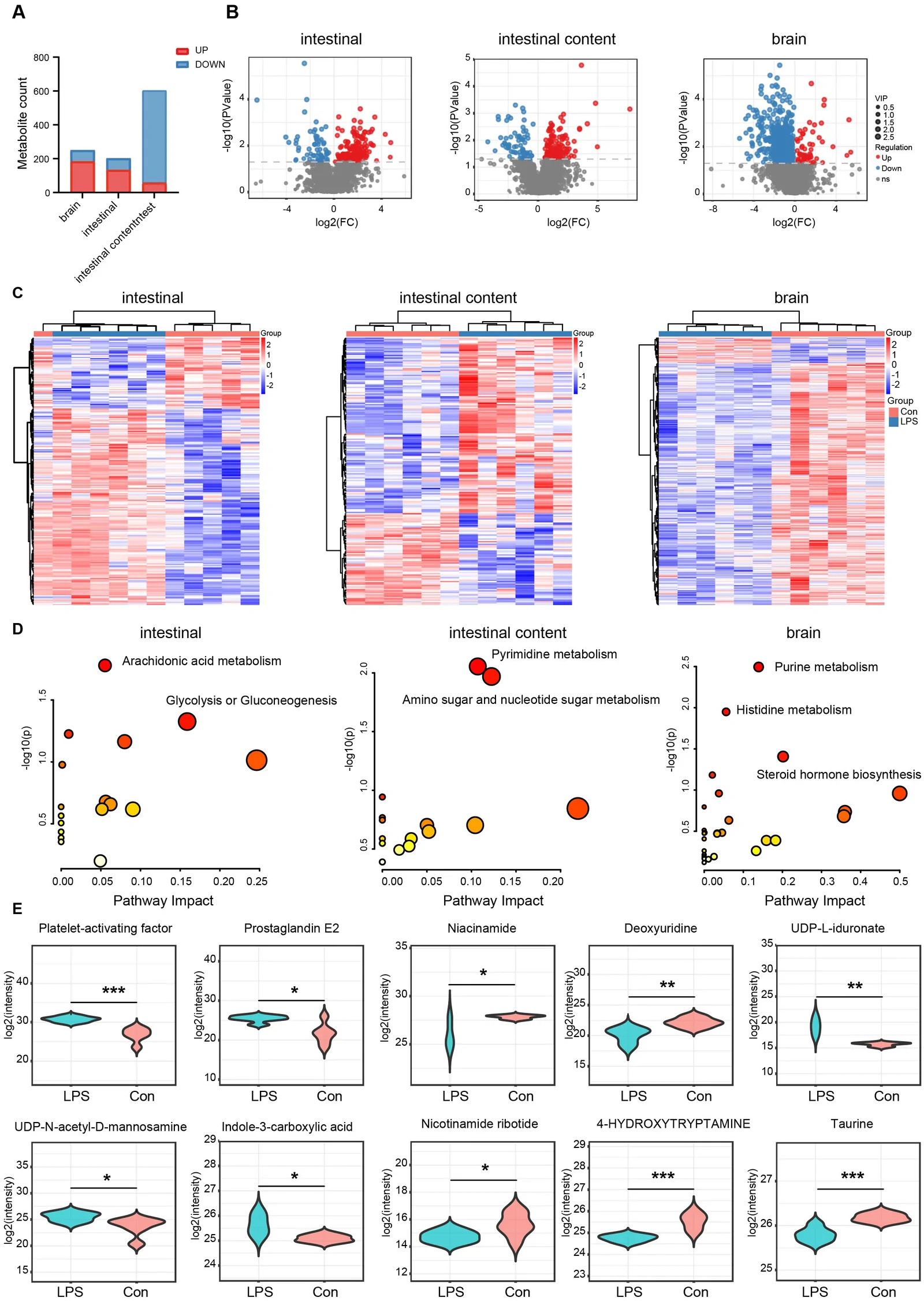
Differential metabolite analyses between the LPS and control groups in brain tissue, intestinal tissue, and intestinal contents showing **(A)** bar plots of differential metabolite expression, **(B)** volcano plots of differential metabolites, **(C)** hierarchical clustering heatmaps of differential metabolites, **(D)** bubble plots of KEGG enrichment analysis, and **(E)** bar plots of key differential metabolites.

A total of 249 significant differential metabolites were identified in intestinal tissue, including 183 upregulated and 66 downregulated metabolites (**Figure 6A**). Compared with the control group, the levels of arachidonic acid-derived inflammatory lipids, including platelet-activating factor (PAF; log2FC = 3.43, p = 5.86 × 10^^-^4) and prostaglandin E2 (PGE2; log2FC = 1.89, p = 0.016), were significantly increased in the LPS group (**Figures 6B, C**; **Supplementary Table 3**). In addition, pathway enrichment analysis showed that arachidonic acid metabolism and glycolysis or gluconeogenesis were significantly altered (**Figure 6D**).

In intestinal contents, 201 significantly differential metabolites were identified, of which 132 were upregulated and 69 were downregulated (**Figure 6A**). In the LPS group, the NAD+ precursor niacinamide (log2FC = -1.26, p = 0.0226) and pyrimidine metabolism intermediates, including deoxyuridine (log2FC = -1.94, p = 0.00545) and thymine (log2FC = -1.54, p = 0.048), were significantly decreased. By contrast, multiple amino sugar and nucleotide sugar intermediates were significantly increased, such as UDP-L-iduronate (log2FC = 4.12, p = 0.00245) and UDP-N-acetyl-D-mannosamine, suggesting activation of mucopolysaccharide and glycoprotein biosynthetic pathways. In addition, indole-3-carboxylic acid was significantly increased (log2FC = 0.59, p = 0.0209), indicating possible disturbance of tryptophan metabolism (**Figures 6B, C**; **Supplementary Table 4**). Pathway enrichment analysis showed significant changes in pyrimidine metabolism and amino sugar and nucleotide sugar metabolism (**Figure 6D**).

In brain tissue, a total of 603 significantly differential metabolites were identified between the two groups, including 57 upregulated and 546 downregulated metabolites (**Figure 6A**), indicating that LPS induced an overall “depletion” state in brain metabolism. In addition, our results showed that multiple neuroprotective or neurotransmitter-related metabolites were significantly downregulated, including taurine (log2FC = -0.38, p = 0.00049) and 4-hydroxytryptamine (log2FC = -0.82, p = 0.00073), as well as several histidine derivatives such as N-acetylhistidine, L-histidinol-phosphate, and N,N-dimethylhistidine; the purine metabolism intermediates 5-methylthioadenosine (MTA) and isopentenyl adenosine; and redox-related metabolites such as S-(formylmethyl)glutathione and nicotinamide ribotide (NMN) (**Figures 6B, C**; **Supplementary Table 5**). To further explore the major metabolic pathways involved, pathway enrichment analysis of the identified differential metabolites was performed. The results showed that these differential metabolites were mainly enriched in purine metabolism, histidine metabolism, and steroid hormone biosynthesis (**Figure 6D**). Taken together, these findings indicate that LPS exposure was associated with reduced levels of several nucleotide- and NAD+-related metabolites in intestinal contents, increased inflammatory lipid mediators in intestinal tissue, and widespread metabolic alterations in the brain.

### 3.6 Transcriptomic analysis reveals LPS-induced alterations in the zebrafish brain gene expression profile

**Figure 7:**
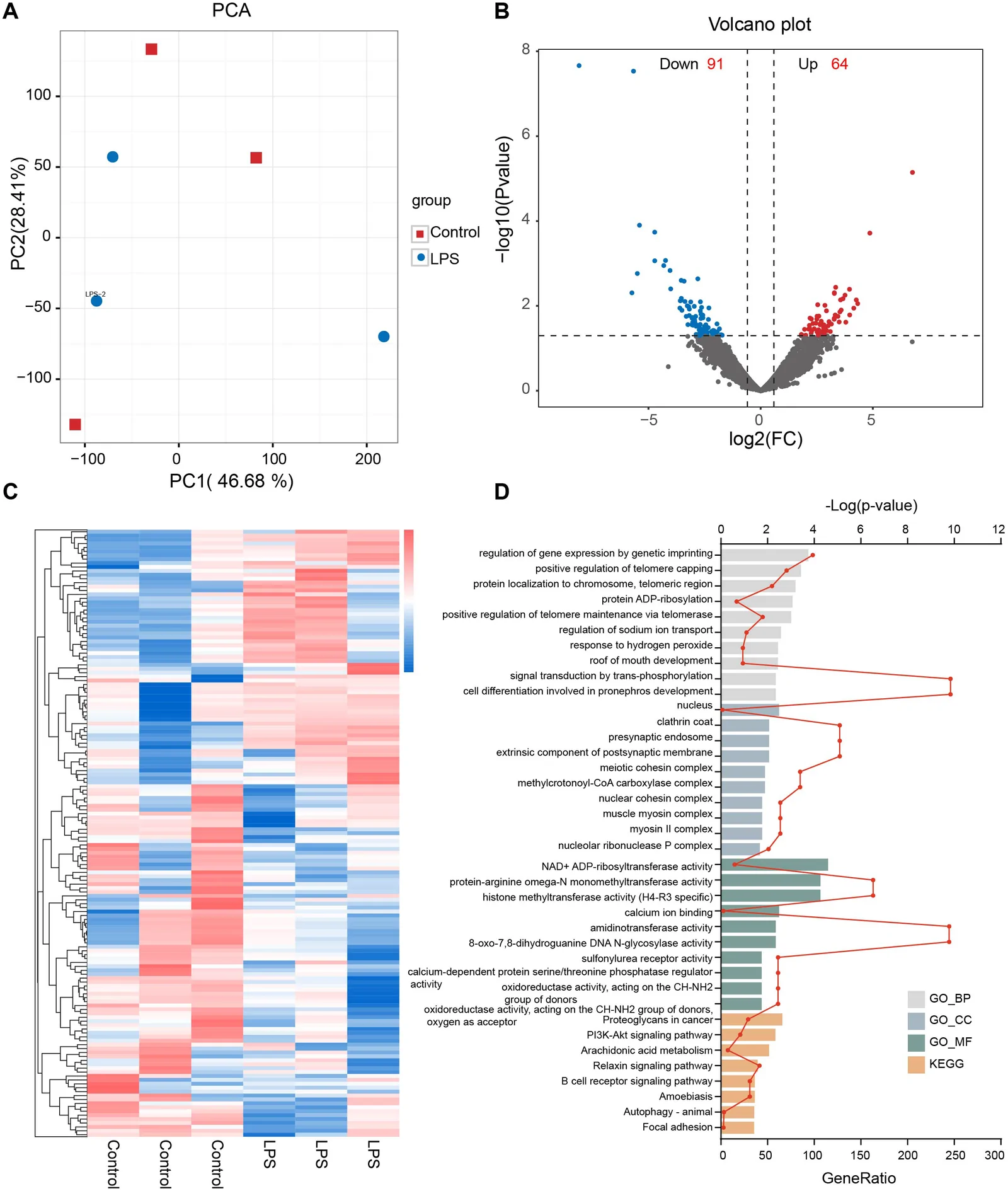
LPS treatment markedly altered the transcriptomic profile of zebrafish brain tissue. Comparison between the LPS and control groups showing **(A)** PCA plot, **(B)** volcano plot of differentially expressed genes, **(C)** clustered heatmap of differentially expressed genes, and **(D)** bar plots of GO and KEGG enrichment analyses.

RNA sequencing was next performed on brain tissues from the control and LPS groups to examine the effect of LPS on the zebrafish brain transcriptome. PCA showed partial separation between the LPS and control groups, with PC1 and PC2 explaining 46.68% and 28.41% of the total variance, respectively. However, appreciable inter-individual variability was observed, with one LPS sample displaying a transcriptional profile relatively closer to the control samples (**Figure 7A**). A similar pattern was observed in the hierarchical clustering analysis (**Figure 7C**), suggesting heterogeneous transcriptional responses to LPS exposure among individual zebrafish. Differential gene expression analysis based on DESeq2 identified 155 DEGs using the criteria |log2FC| ≥ 1 and p < 0.05, including 91 downregulated and 64 upregulated genes. A volcano plot of the DEGs is shown in **Figure 7B**. Among these genes, the antioxidant-related glutathione peroxidase *gpx1a-2*, the hypoxia-inducible factor *hif1al2*, and the telomere-related genes *tnksa/tnksa-3* were significantly upregulated, suggesting activation of oxidative stress and a pseudohypoxic state in the brain, together with a telomere-protective response. By contrast, the DNA repair enzyme *parp8*, prostaglandin E synthase *ptges3a-2*, and the PI3K adaptor *pik3ap1–2* were significantly downregulated, implying suppression of DNA damage repair and some lipid signaling pathways (**Figure 7C**; **Supplementary Table 6**).

Functional enrichment analysis of the identified DEGs showed significant enrichment in 252 biological process (BP) terms, with the top three being “regulation of gene expression by genetic imprinting,” “positive regulation of telomere capping,” and “protein localization to chromosome, telomeric region.” In the cellular component (CC) category, 19 terms were significantly enriched, with the top three being “nucleus,” “clathrin coat,” and “presynaptic endosome.” In the molecular function (MF) category, 29 terms were significantly enriched, including “NAD+ ADP-ribosyltransferase activity,” “protein-arginine omega-N monomethyltransferase activity,” and “histone methyltransferase activity (H4-R3 specific)” (**Figure 7D**). KEGG pathway enrichment analysis showed that the DEGs were significantly enriched in the PI3K-Akt signaling pathway, arachidonic acid metabolism, autophagy - animal, and focal adhesion (**Figure 7D**). Among these, PI3K-Akt and focal adhesion are closely related to neuronal survival, synaptic plasticity, and BBB integrity, whereas enrichment of arachidonic acid metabolism was highly consistent with the arachidonic acid-related alterations observed in the gut and brain metabolomes.

### 3.7 Cross-omics correlation analysis identifies potential associations among gut microbiota, metabolites, and brain gene expression

**Figure 8:**
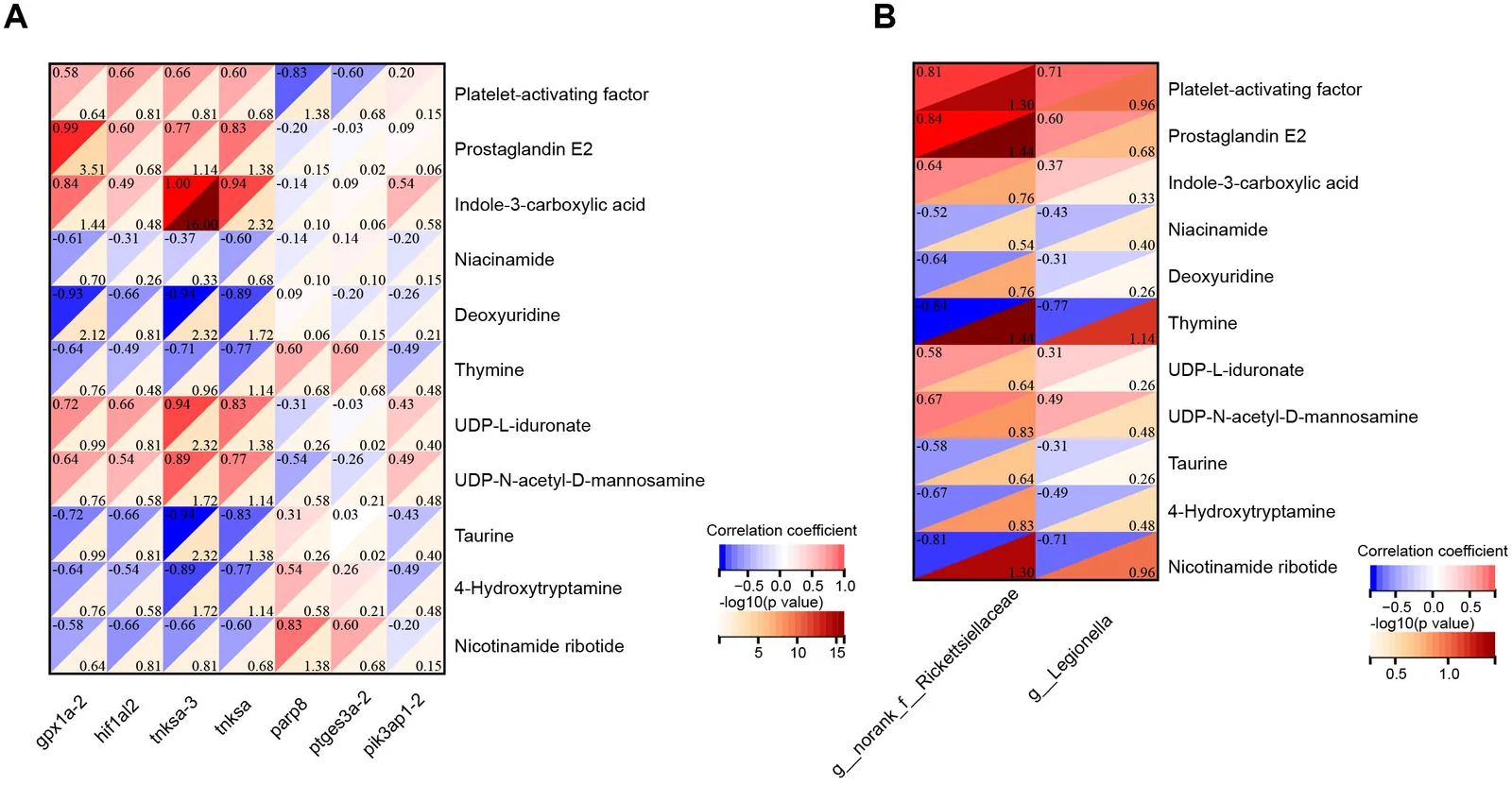
**(A)** Spearman correlation heat map between key differential metabolites and differentially expressed genes. **(B)** Correlation heat map between key differential metabolites and differential bacterial genera.

To further investigate potential associations among differential gut microbes, metabolites, and gene expression levels, correlation analyses were performed. Analysis of key differential metabolites in intestinal tissue, intestinal contents, and brain tissue together with key genes in the brain showed that *gpx1a-*2 expression was positively correlated with PGE2 levels, and *tnksa-3* expression was positively correlated with indole-3-carboxylic acid levels; both genes were negatively correlated with deoxyuridine and thymine levels (**Figure 8A**). Correlation analysis between key differential metabolites and differential gut microbiota at the genus level further showed that an unclassified genus within the family *Rickettsiellaceae* and the genus *Legionella* were positively correlated with platelet-activating factor and prostaglandin E2 levels, but negatively correlated with thymine and nicotinamide ribotide levels (**Figure 8B**).

## 4 Discussion

In this study, we established an endotoxemia-like systemic inflammation model in zebrafish by intraperitoneal injection of LPS and conducted a preliminary investigation and integrative analysis across multiple levels, including behavior, histopathology, live imaging in larvae, gut microbiota, multi-tissue metabolomics, and brain transcriptomics, with the aim of identifying potential links among gut microbiota, microbial metabolites, host metabolism, and host behavior. The results showed that LPS induced intestinal inflammation and mucosal barrier damage in zebrafish, accompanied by gut microbial dysbiosis and increased inflammatory lipid mediators. In the larval model, LPS also caused rapid neutrophil mobilization and cerebral vascular leakage. Further behavioral analyses demonstrated that LPS induced anxiety-like behavior and impaired spatial learning and memory in adult zebrafish. Cross-omics analyses further identified molecular alterations in the brain associated with oxidative stress, autophagy, and adhesion/cytoskeletal pathways, accompanied by widespread metabolic disturbances. Taken together, these findings suggest that LPS-induced systemic inflammation is associated with neurovascular dysfunction, neuroinflammatory responses, and alterations in neurotransmitter and energy metabolism, which may collectively contribute to the observed behavioral abnormalities.

Sepsis-associated systemic inflammation can rapidly affect the neurovascular unit, leading to increased microvascular permeability and impaired BBB function, thereby creating conditions that allow peripheral inflammatory mediators, cells, and metabolites to enter the brain (Su et al., 2025; Yue et al., 2025). In the present study, vascular leakage was observed within a 6-h time window, suggesting that the zebrafish model may be useful for resolving the temporal sequence of early events in SAE and for providing a highly sensitive window for subsequent pharmacological intervention screening.

A recent study highlighted the dual role of the intestine in sepsis, with intestinal hypoperfusion and barrier disruption occurring early in sepsis and gut microbial dysbiosis potentially further amplifying systemic inflammation and organ injury (Chancharoenthana et al., 2023). A previous study reported that sepsis is associated with decreased gut microbial diversity, expansion of opportunistic pathogens, and altered microbial functions, which may be related to inflammatory severity, secondary infection, and clinical outcomes (Sun et al., 2023). In addition, gut microbiota-derived metabolites, including short-chain fatty acids, tryptophan metabolites, bile acids, and lipid mediators, can regulate mucosal immunity and epithelial tight junctions, thereby influencing endotoxin translocation into the circulation and injury to distant organs (Cai et al., 2022; Lou et al., 2022). In the present study, LPS markedly upregulated inflammatory cytokines in intestinal tissue and was accompanied by villus loss and neutrophil infiltration, collectively supporting the occurrence of mucosal inflammation. Tight junction- and mucus barrier-related genes exhibited discordant changes. Together with previous zebrafish enteritis studies reporting mucosal barrier injury, altered mucus secretion, and post-injury repair processes, these findings suggest that the intestinal mucosal barrier may exist in a dynamic state in which injury and compensatory remodeling coexist (Morales Castro et al., 2025; Ni et al., 2023; Zhao et al., 2023). In addition, 16S rRNA sequencing showed that although alpha diversity did not change significantly, overall community structure shifted markedly. At the phylum level, relative abundances of *Fusobacteriota* and *Actinomycetota* decreased, whereas those of *Pseudomonadota* and *Bacillota* increased. LEfSe analysis further identified enrichment of the genus *Legionella* in the LPS group, suggesting that LPS-associated intestinal inflammation was accompanied by alterations in specific bacterial taxa. Previous studies have shown that *Legionella* is a typical opportunistic pathogen that can persist in complex aquatic environments and protozoan hosts and cause disease through intracellular replication (Gonçalves et al., 2021; Nisar et al., 2020). Moreover, zebrafish infection models have demonstrated that *Legionella* can induce dose-dependent mortality in larvae and recruit macrophages and neutrophils to host defense (Ayesha et al., 2023; Viana et al., 2023). These findings suggest that LPS-induced intestinal barrier disruption and systemic inflammation may contribute to gut microbiota imbalance, expansion of opportunistic pathogens, and subsequent perturbation of host inflammatory and metabolic homeostasis through microbial metabolites.

At the metabolomic level, compared with the control group, the inflammatory lipid mediators PAF and PGE2 were significantly increased in intestinal tissue in the LPS group, indicating that arachidonic acid-related inflammatory lipid pathways were activated after LPS exposure. PGE2 is an important lipid mediator generated from arachidonic acid by cyclooxygenases (COX-1/2). It not only participates in the regulation of intestinal inflammatory responses but can also affect mucosal immune homeostasis and epithelial barrier repair through receptors such as PTGER4 (Martínez-Colón and Moore, 2018; Na et al., 2021). PAF is an important phospholipid mediator linking inflammatory responses and platelet activation (Lordan et al., 2021) and can activate pro-inflammatory pathways such as NF-κB/IL-8 in intestinal epithelial cells (Borthakur et al., 2010). Therefore, the simultaneous increase in PAF and PGE2 observed in this study suggests that these mediators may jointly participate in amplification of LPS-induced intestinal inflammation and further affect intestinal barrier function, immune cell activation, and intestinal physiological homeostasis. These metabolomic findings are consistent with a sustained pro-inflammatory intestinal environment accompanied by barrier alterations and metabolic disturbance following LPS exposure. Our data also showed that LPS decreased niacinamide and pyrimidine metabolism intermediates in intestinal contents, suggesting insufficient supply of nucleotides and NAD+ precursors. These changes may reflect altered luminal nutrient availability, intestinal absorption, or microbial metabolic activity. Their potential relationship with cerebral energy and redox homeostasis remains speculative and requires further investigation. Notably, most metabolites in the brain were significantly decreased in the LPS group, including purine and histidine metabolism intermediates, neurotransmitter-related metabolites, and NAD+-related metabolites. Because SAE is commonly accompanied by mitochondrial dysfunction, oxidative stress, and neurotransmitter imbalance (Sekino et al., 2022), the reductions in neuroprotective or neurotransmitter-related molecules such as taurine and 4-hydroxytryptamine observed here are consistent with the anxiety-like behavior and learning/memory impairment. Taken together, these metabolic alterations suggest disturbances in cerebral energy, redox, and neurotransmitter-related metabolism. Such changes may be relevant to the observed neurobehavioral abnormalities, although their effects on synaptic plasticity and neural circuit function were not directly examined in the present study.

Brain transcriptome results provided further evidence at the molecular signaling level. Upregulation of *gpx1a-2* and *hif1al2* indicates that oxidative stress and hypoxic responses were significantly activated (Handy and Loscalzo, 2022; Yfantis et al., 2023). Significant enrichment of the PI3K-Akt, focal adhesion, autophagy, and arachidonic acid metabolism pathways indicates that neuronal survival regulation, cell adhesion/cytoskeletal maintenance, autophagic responses, and lipid inflammatory pathways were all markedly altered in brain tissue after LPS exposure. The PI3K-Akt and focal adhesion pathways are closely associated with neuronal survival regulation, maintenance of synaptic homeostasis, and structural integrity of the neurovascular unit/BBB (Orr et al., 2022; Zhou et al., 2024). These transcriptomic findings are consistent with the cerebral vascular leakage phenotype observed in the larval model and suggest potential involvement of neurovascular dysfunction in the SAE-like phenotype.

Cross-omics correlation analyses identified potential associations among gut microbial alterations, inflammatory lipid profiles, nucleotide/NAD+-related metabolism, and brain stress-related molecular responses. Differential bacterial genera were positively correlated with pro-inflammatory lipid levels and negatively correlated with energy-related metabolites such as thymine and NMN. Increased PGE2 was also closely associated with upregulation of antioxidant genes in the brain, such as *gpx1a-2*. These associations suggest potential links among gut microbial alterations, inflammatory lipid metabolism, energy-related metabolites, and cerebral stress responses. Although these correlation analyses provide important clues, their causal relationships still require experimental validation.

However, this study also has certain limitations. First, the proposed relationships among gut microbial alterations, metabolic changes, and brain-associated phenotypes are based primarily on correlational evidence and lack direct causal validation. Second, the metabolomics analysis was untargeted, and key metabolites still require targeted quantitative validation to determine their absolute levels and temporal dynamics, as well as to further clarify their receptors and downstream pathways. Third, the 16S rRNA gene sequencing approach used in this study provides limited taxonomic resolution for *Legionella* and does not allow reliable species- or strain-level identification or direct functional characterization. Therefore, the observed enrichment of *Legionella* should be interpreted as a genus-level association rather than evidence for a specific pathogenic species or functional mechanism. Future studies using shotgun metagenomic sequencing and targeted species-level validation will be required to clarify its potential functional relevance. Fourth, the transcriptomic analysis was performed with a relatively small number of biological replicates, which may reduce statistical power and increase the influence of inter-individual variability. Consistent with this limitation, PCA and hierarchical clustering revealed heterogeneous transcriptional responses within the LPS group, with some overlap between LPS and control samples. Therefore, the transcriptomic findings and subsequent pathway-enrichment analyses should be regarded as exploratory and interpreted cautiously. Further validation in larger independent cohorts and by targeted approaches, such as RT-qPCR, will be necessary to confirm these findings. Fifth, the cross-omics analyses in the present study were based on correlations among selected differential features and therefore should be regarded as exploratory rather than as a comprehensive integrative multi-omics model. These associations do not establish direct functional or causal relationships and require validation using larger, matched cohorts and dedicated multi-omics integration approaches. In addition, comprehensive sample-level multi-omics integration approaches such as DIABLO were not applied because the transcriptomic, metabolomic, and microbiome datasets were not generated from fully matched biological samples and differed in sample size. Future studies using larger cohorts with matched multi-omics measurements from the same individuals will enable more robust simultaneous integration across omics layers.

## 5 Conclusion

In summary, this study established an LPS-induced endotoxemia-like inflammatory model in zebrafish and systematically demonstrated, through evidence from behavioral assessment, histopathological observation, larval *in vivo* imaging, and multi-omics profiling, LPS exposure was associated with intestinal inflammation and barrier injury, gut microbiota remodeling, metabolic disturbances, alterations in brain signaling pathways, and neurobehavioral abnormalities in zebrafish. Cross-omics analyses further identified potential associations among these changes, suggesting that disruption of the microbiota–gut–brain axis may be involved in LPS-induced SAE-like phenotypes. At a systems level, these findings provide preliminary insights into the potential involvement of the microbiota–gut–brain axis in SAE-like phenotypes and offer a basis for further mechanistic investigation and validation in complementary experimental models.

## Data Availability

The datasets presented in this study can be found in online repositories. The names of the repository/repositories and accession number(s) can be found below: https://www.ncbi.nlm.nih.gov/, PRJNA1418344.
